# Mesenchymal stromal cells modulate neutrophil phenotype via paracrine signals

**DOI:** 10.1186/s13287-025-04684-w

**Published:** 2025-10-21

**Authors:** Laudy Cherry, Sinziana Popescu, Carmen Alexandra Neculachi, Evelyn-Gabriela Nastase-Rusu, Catalina Iolanda Marinescu-Colan, Bogdan Paul Cosman, Letitia Ciortan, Fabio Martelli, Maya Simionescu, Elena Butoi, Alexandrina Burlacu, Mihai Bogdan Preda

**Affiliations:** 1 Institute of Cellular Biology and Pathology “Nicolae Simionescu”, Romanian Academy, 050568 Bucharest, Romania; 2 Molecular Cardiology Laboratory, IRCCS Policlinico San Donato, San Donato Milanese, 20097 Milan, Italy

**Keywords:** Mesenchymal stromal cells, Neutrophils, Myocardial infarction, Remote cell therapy, Inflammation

## Abstract

**Background:**

Despite the recognized importance of neutrophils in cardiac repair following myocardial infarction (MI), their interaction with mesenchymal stromal cells (MSCs), particularly regarding polarization phenotypes and functional impacts, remains unclear. Here, we investigated these interactions across controlled in vitro systems and an in vivo MI model.

**Methods:**

Human HL-60 cells were differentiated into neutrophil-like cells (dHL-60) and polarized toward N1/N2 states to test the MSC paracrine effects using indirect transwell coculture. Readouts included gene expression, cytokine profiling and functional assays. To increase translational relevance, indirect ex vivo cocultures of human MSCs with primary neutrophils isolated from MI mice or from MI patients were analysed for gene expression and cytokine profiles in conditioned media. In vivo, syngeneic mouse MSCs were transplanted subcutaneously immediately after MI, and early cardiac function was evaluated by echocardiography. Cardiac neutrophils where quantified by flow cytometry, and Ly6G^+^ neutrophils from infarcted hearts and peripheral blood were purified by MACS for bulk RNA-seq with targeted RT-qPCR validation.

**Results:**

In vitro, MSCs suppressed pro-inflammatory mediators in N1-like neutrophils and enhanced reparative factors in N2-like cells. In vivo, remote MSC transplantation improved early cardiac performance, and reduced neutrophil accumulation in the infarct. Paradoxically, cardiac neutrophils showed transcriptomic enrichment of inflammatory pathways, whereas blood neutrophils showed reduced interferon-related programs.

**Conclusion:**

Our findings indicate that MSCs can modulate neutrophil responses, underscoring the nuanced effects of MSC-based approaches in ischemic heart disease. These results suggest that anti-inflammatory effects observed under controlled conditions may not fully translate in vivo, highlighting the importance of context when evaluating MSC-based therapies.

**Supplementary Information:**

The online version contains supplementary material available at 10.1186/s13287-025-04684-w.

## Background

Stem cell research remains a central pillar in advancing therapeutic strategies for ventricular remodelling following ischemic cardiac injury. Although substantial progress has been made, the perception of stem cells as a simple, “off-the-shelf” solution often misrepresents the intricacies of this field. The reality is more complex, and the researchers continue to investigate the full therapeutic potential of stem cells in slowing disease progression. Mesenchymal stromal cells (MSCs) have gained significant attention for their potential clinical applications. However, their direct integration into host tissue remains limited, with paracrine immunomodulatory mechanisms being extensively investigated through the past years. Previous studies have highlighted challenges such as unpredictable therapeutic outcomes due to cell death following local transplantation into injured tissue [1–3]. Moreover, MSC priming, intended to mimic the post-transplantation microenvironment or enhance their therapeutic effects, can be detrimental, particularly with prolonged duration or combined treatments [4, 5].

Ischemic injury triggers inflammation, leading to myeloid cell infiltration into the heart after myocardial infarction (MI) in overlapping waves of neutrophils and monocytes [6]. The intensity and duration of this inflammatory response are crucial in determining the trajectory of heart failure, with evidence underscoring the importance of mitigating inflammation to enhance cardiac repair [7]. Intriguingly, recent findings suggest that the prevailing model of myeloid cell specialization within the injured tissue microenvironment may be incomplete. Instead, evidence indicates that innate immune pathways are activated at distant sites before these cells infiltrate infarcted hearts in both mice and humans [8, 9].

Prior observations indicates that MSCs do not need to directly home to the injury site to achieve therapeutic benefits in acute MI [10]. Instead, even when administered remotely, such as through subcutaneous transplantation, MSCs can induce systemic effects. This occurs through a transient release of circulating factors, triggered by local hypoxic conditions, which may modulate inflammatory and reparative responses at distant target tissues [11].

Despite limited understanding of the mechanisms that regulate MSC migration and survival after transplantation, their immunomodulatory effects predominantly involve paracrine signalling, influencing both innate and adaptive immunity [12]. While extensive research has investigated the crosstalk between MSCs and macrophages, and particularly MSCs’ capacity to polarize macrophages into distinct immune phenotypes [13, 14], the interactions between MSCs and neutrophils, particularly critical in the early inflammatory phase, remain insufficiently explored.

We hypothesized that MSCs could remotely modulate neutrophil phenotype and function, thereby influencing post-MI inflammation and repair. To address this, we performed in vitro and in vivo experiments using murine and human cells and a mouse MI model to characterize MSC-neutrophil interactions. In vitro, MSCs promoted a reparatory/N2 neutrophil phenotype via paracrine factors. However, in vivo experiments revealed greater complexity: remote MSC transplantation reduced neutrophil infiltration into infarcted myocardium but induced an inflammatory transcriptomic signature in cardiac neutrophils, underscoring context-dependent MSC-neutrophil interactions.

## Methods

### Animal studies

Mice were housed under controlled environment with a 12/12-h light/dark cycle, 21 °C, 55–60% humidity, and access to chow and water ad libitum. All animal procedures were performed in accordance with the ARRIVE guidelines 2.0. Single animals were used as experimental unit for in vivo experiments, unless otherwise stated. Three groups of animals were used: control animals (Sham-operated), animals with MI and animals with MI treated with MSC.

MI was induced by left coronary artery (LCA) ligation, as previously described [15]. In brief, 12- to 16-week-old C57Bl/6J male mice were anesthetized with ketamine/xylazine (100/20 mg/kg) and intubated orotracheally. A left thoracotomy was performed in the fourth intercostal space to expose the heart, and the LCA was ligated using a 7 − 0 silk suture. The chest and muscle layers were closed with 6 − 0 polypropylene sutures, and the mice were allowed to recover on a 37 °C heating plate. Buprenorphine hydrochloride (Temgesic^®^, 0.1 mg/kg) was administered subcutaneously immediately and 24 h after surgery for analgesia. Syngeneic mouse MSCs were isolated from the bone marrow of 6–8-week-old male C57Bl6 mice, as previously described [11, 16, 17], and used between passages 6–9. MSCs were thawed (5 × 10^3^ cells/cm^2^) and cultured in low-glucose DMEM supplemented with 10% MSC-qualified FBS (Gibco, 12662029) for 5 days to reach confluence before being used for transplantation. Within the first thirty to sixty minutes following LCA ligation, mice received 3 subcutaneous injections of 10⁶ MSCs (in 50 µL PBS/site) at different sites: interscapular, dorsal and inguinal. After 3 days, the animals were re-anesthetized and blood was collected by cardiac puncture before heart harvesting. Only animals with MI confirmed by echocardiography were included. Mice in which MI could not be confirmed (or that developed perioperative complications) were excluded from further analyses and managed according to predefined humane endpoints. No animals were excluded from the analysis after initial validation. The investigators were not blinded to the design of the study or during data collection and analysis.

### Heart digestion

The hearts were collected in 5 ml ice-cold PBS, and the left ventricles below the ligature were used for digestion. The tissue was finely minced with a scalpel in a Petri dish kept on ice and then transferred to a C-tube for the gentleMACS™ Octo Dissociator (Miltenyi Biotec, 130-093-237). After sedimentation, the tissue was washed with 5 ml cold PBS. Next, 5 ml of enzyme solution (Liberase DH at 25 µg/ml and DNase I at 40 µg/ml in PBS) at 37 °C was added to the tissue fragments. The gentleMACS Octo Dissociator protocol had the following steps: (i) Ramp 10 s x 300 rpm; Temp ON; (ii) Spin 1 min x 20 rpm; (iii) 40 cycles of: -250 × 20 s; +250 × 20 s; Stop. Cell homogenate was passed through a 21G needle 2–3 times, mixed with 10 ml cold DMEM + 10% fetal bovine serum (FBS), and passed through a 40 μm strainer into a 50 ml Falcon tube. The tube was centrifuged at 400xg for 5 min at 4 °C, followed by careful removal of the supernatant. The pellet was resuspended in 5 ml of 1x RBC lysis buffer (Miltenyi Biotec, 130-094-183), incubated for 4 min at room temperature in the dark, then washed with 10 ml cold PBS + 10% FBS and centrifuged again at 400xg for 5 min at 4 °C. Finally, the pellet containing cardiac cells was either used for flow cytometry or for magnetic-activated cell sorting (MACS) neutrophil purification.

### Magnetic immunoselection of neutrophils

Cardiac cells obtained after heart digestion were resuspended in 90 µl cold sorting buffer (PBS + 0.5% BSA + 2 mM EDTA). Subsequently, 10 µl anti-Ly6G microbeads (Miltenyi Biotec, 130-120-337) were added. The samples were incubated for 10 min at 4 °C, then 1 ml RT sorting buffer was added, and the suspension was centrifuged at 400xg for 5 min at 4 °C. The pellet was resuspended in 500 µl RT sorting buffer. An MS column was placed in the MACS magnet and rinsed with 500 µl RT sorting buffer. The cell suspension was applied to the MS column, and the effluent (Ly6G-negative cells) was collected. The column was washed three times with 1500 µl RT buffer, and gentle pressure was applied with a plunger to avoid flow blockages and ensure thorough rinsing. The column was removed from the magnet, and 1 ml of buffer was used with a plunger to collect Ly6G-positive cells. The cells were then centrifuged at 400xg for 5 min at 4 °C, and the pellet resuspended in 500 µl Trizol and stored at -80 °C.

### Cell culture

The HL-60 and THP-1 cell lines were purchased from ATCC (#CCL240™, #TIB-202™). Cells were routinely cultured in Iscove’s Modified Dulbecco’s Medium (IMDM, Gibco 31980-030) supplemented with 20% FBS (Gibco, A5256701) for HL-60 cells, or RPMI-1640 Medium (Gibco, A1049101) supplemented with 0.05 mM 2-Mercaptoethanol + 10% FBS, and maintained through periodic splitting according to the vendor’s protocols. Human MSCs were isolated from bone marrow as previously described [15] and subcultured at a density of 5000 cells/ cm^2^, in 1 g/L glucose Dulbecco’s Modified Eagle Medium (DMEM, Gibco, 10567014) supplemented with 10% MSC-qualified FBS (Gibco, 12662029). Human MSCs were used between the 6th and 9th passages.

### Cell differentiation and polarization protocol

Pro-myeloblasts (HL-60 cell line) and monocytic cells (THP-1 cell line) were used for in vitro generation of N0 neutrophils and M0 macrophages, respectively. To differentiate HL-60 cells towards a neutrophil-like phenotype, 1.3% DMSO was added to the cell suspension and then cultivated for 5 days in complete media (IMDM + 20% FBS). The resulting differentiated HL-60 (dHL-60) cells were assessed for proliferation arrest, nuclear segmentation and expression of specific differentiation markers. To differentiate THP-1 cells towards a M0 macrophage phenotype, 100 nM PMA was added into the cell suspension for three days. To induce N1/N2 polarization of dHL-60, the cells were further cultivated for 48 h in complete medium supplemented with 100ng/ml hu IFNγ (SRP3058, Sigma-Aldrich) and 100ng/ml LPS (L2630, Sigma-Aldrich) for N1 and 20ng/ml hu IL-4 (SRP3093, Sigma-Aldrich) for N2. Similar treatments were applied for THP-1-derived M0 macrophages to obtain M1/M2 macrophages. Assessment of specific differentiation markers was performed by subsequent qPCR analysis.

### Indirect transwell coculture experiments

HL-60 cells were differentiated to neutrophil-like cells with 1.3% DMSO for 5 days. On day 5, dHL-60 were cocultured with human MSCs using an indirect coculture system (ThermoFisher Scientific, 140640). 0.4-µm transwell inserts containing 3 × 10^6^ dHL-60 in coculture medium (IMDM + 20% FBS) were placed above the human MSCs (seeded the day before at a density of 3 × 10^5^ cells/well in the lower chamber of the plate), and cocultured for 48 h. Polarizing stimuli toward N1 (LPS + IFNγ) or N2 (IL-4) were maintained during coculture to preserve phenotype. dHL-60 were collected for RNA isolation/RT-qPCR, and supernatants were retained for cytokine assays when indicated.

Primary blood neutrophils were cocultured 24 h with human MSCs using the same system as above. For murine model, on day 3 post-MI, blood was processed and Ly6G + neutrophils were purified by MACS. Transwell inserts were loaded with 0.7–1 × 10^6^ murine neutrophils (pooled from 2 animals) and cocultured with 3 × 10^5^ human MSCs per well. After 24 h incubation, neutrophils were harvested for RT-qPCR, and supernatants were clarified (400×g, 5 min, 4 °C, and 2000×g, 30 min, 4 °C) and analysed with the Mouse XL Cytokine Array (R&D Systems). For human model, peripheral blood was obtained from two MI patients during the first 24 h after infarction, under approved ethics and written consent. Human polymorphonuclear cells (hPMN) were enriched by density gradient centrifugation. 9 × 10^5^ cells/insert were cocultured 24 h in 0.4-µm transwells with 3 × 10^5^ human MSCs per well. After 24 h incubation, hPMN were pooled from 2 wells per experimental replicate and processed for RT-qPCR. PMN-only wells served as controls.

### Flow cytometry

HL-60 and dHL-60 cells were resuspended at 10^6^ cells/mL in FACS buffer (PBS containing 2% FBS). For each analysis, 100 µL cell suspension was incubated with an appropriate dilution of anti-CD11b/APC (Beckman Coulter, A87782), anti-CD18/APC (Biolegend, 373406), or isotype-matched control fluorescent antibody for 30 min on ice, then washed and centrifuged at 400 × g for 5 min. The cell pellets were then resuspended in 250 µL FACS buffer in the presence of propidium iodide (2 µg/mL final concentration) and analysed by flow cytometry.

Similarly, 100 µl of cardiac cell suspensions (10^6^ cells/ml FACS buffer) were incubated on ice for 30 min with the following antibodies: anti-CD45-PE, clone 30-F11 (BioLegend 103106), anti-CD11b-APC/Cy7, clone M1/70 (BioLegend 101226), anti-Ly-6 C-FITC, clone HK1.4 (Biolegend 128006) and anti-Ly-6G-BV421 clone 1A8 (Biolegend 127627). Cells were thereafter washed and resuspended for flow analysis. Flow cytometry acquisition was performed using a CytoFlex cytometer (Beckman Coulter, Indianapolis, IN, USA) and the data were analysed with CytExpert 2.5 software (Indianapolis, IN, USA). For flow cytometry in MI experiments, *n* = 2 (sham) or 4 (MI and MI + MSC) animals per group were used.

### Nuclear morphology

dHL-60 cells were seeded on a standard microscope slide using a cytocentrifuge (Hettich Cyto-System) and then Giemsa staining protocol was performed according to the manufacturer’s specifications (Merck, 48900-100ML-F). Representative images of stained dHL-60 cells were captured with a Leica DMi8 inverted microscope equipped with an DFC450-C colour camera and controlled via LAS X software (Leica Microsystems) using a PLAN APO 100x/1.40 oil immersion objective.

### Quantitative real-time RT-PCR

Total RNA was purified from human cell samples using the PureLink RNA Mini Kit (Invitrogen, Waltham, MA, USA) and the concentrations were estimated based on the absorbance at 260 nm using the Nanodrop ND-1000 Spectrophotometer (Marshall Scientific, Hampton, NH, USA). Reverse transcription was performed using the High-Capacity cDNA Reverse Transcription Kit (Cat. No: 4368814, Applied Biosystems) according to the manufacturer’s instructions, starting from 0.4 µg total RNA per reaction. Real-time PCR was performed using Platinum™ Taq DNA Polymerase (Cat. No: 10966083, Invitrogen, Waltham, MA, USA) on a 7900HT Fast Real-Time PCR System (Applied Biosystems). Human-specific primers were designed using NCBI Primer-BLAST and the list of primer sequences is given in the Table S1. Data analysis was performed using the comparative CT method, and the normalization was made by reference to Glyceraldehyde 3-phosphate dehydrogenase (GAPDH), Ribosomal Protein L13a (RPL13A), Ribosomal Protein S18 (RPS18) and beta-actin (ACTB).

### Neutrophil activation assay

A total of 2.5 × 10⁵ N0/N1/N2 dHL-60 cells, obtained from either monoculture or MSC coculture, were seeded in poly-L-lysine-coated wells of Seahorse XFp Cell Culture Miniplates using DMEM XF medium (Agilent). Oxygen consumption rate (OCR) was measured both at basal levels and following the addition of 50 nM PMA (P8139, Sigma-Aldrich) to investigate the impact of MSC paracrine factors on neutrophil activation in a non-invasive manner. The experiment was conducted four times in duplicate for each condition.

### Neutrophil phagocytosis assay

HL-60 cells were differentiated and polarized towards N1/N2, as described above, then seeded in 96-well plates at the density of 10^5 cells/well. Next, pHrodo™ Green S. aureus BioParticles™ were resuspended in IMDM + 20% FBS and added to the well, at a concentration of 100 µg/ml. The plates were then centrifuged 300xg, 2 min, RT and incubated for 3 h at 37 °C. At the end, the cells were then resuspended by gentle pipetting, then analysed by flow cytometry in the presence of 10 µg/ml propidium iodide as a viability stain.

### Echocardiography

Transthoracic echocardiography was performed 48 h after LCA ligation using a Vevo 2100 system (VisualSonics) with a 30-MHz MicroScan transducer. Mice were lightly anesthetized with 1–2% isoflurane and maintained at 37 °C. Heart rate was kept within the physiological range (> 400 BPM) by adjusting anesthesia. Two-dimensional B-mode images were acquired in parasternal long-axis (PSLAX) and left ventricle function was calculated from two complete cardiac cycles and included measurements of ejection fraction (EF), fractional shortening (FS), cardiac output (CO), stroke volume (SV), and LV volumes.

### Proteome profiler cytokine array

The cytokine profile of the dHL-60 secretomes before and after N1/N2 polarization was analysed using the Proteome Profiler Human XL Cytokine Array Kit (ARY022B, R&D Systems) following the manufacturer’s instructions. Briefly, the culture medium was collected after 48 h of polarization, centrifuged 400xg/5minutes/RT, then supernatant centrifuged again at 2000xg/30minutes/4°C to collect the conditioned medium, which was thereafter stored at -80 °C. In parallel, the secretome of murine cardiac neutrophils was profiled. Ly6G⁺ neutrophils were isolated from infarcted hearts at day 3 post-MI, and in vitro cocultured with human MSCs for 24 h. Condition media was subsequently analysed using the Proteome Profiler Mouse XL Cytokine Array (ARY028, R&D Systems) according to the manufacturer’s protocol. Arrays were developed by chemiluminescence reaction, and the detection was performed using a Luminescent Image Analyzer LAS-3000 (FUJIFILM, Japan). The pixel densities of all duplicate spots were determined using CLIQS 1D Pro software (TotalLab, United Kingdom).

### RNA isolation, sequencing workflow and bioinformatic analysis

Total RNA was extracted from mouse neutrophils using the Trizol Plus RNA Purification Kit (Thermo Fisher Scientific, 12183555), following the manufacturer’s protocol. RNA-seq was performed by Novogene (UK) Company Limited, following their standard protocols. Library preparation included ribosomal RNA depletion, RNA fragmentation and cDNA synthesis. Followed steps involved end-repair, poly-A tailing and adaptor ligation. Finally, libraries were sequenced as stranded paired-end reads on the Novaseq 6000 platform.

Data processing was performed in-house using a combination of pipelines based on well-established, externally developed bioinformatic tools [18]. The quality of raw sequencing data was checked using FastQC (v0.12.0) followed by adaptor trimming and PCR duplicate removal which were done with Trimmomatic (v0.39) and Picard, respectively. Trimmed reads were then mapped to mouse genome GRCm39 P110 using STAR (v2.7.11), alignment quantification quality was assessed using Samtools (v1.18) while stranded quantification was performed with featureCounts (v2.0.3). Given the relative high percentage of mitochondrial RNA in the dataset, reads mapping to mitochondrial regions were filtered out to mitigate the risk of data bias in downstream analysis. Finally, differential expression analysis was carried out with DESeq2 (v1.44.0), genes were considered significantly expressed with a log2 fold change of ± 0.5 and false discovery rate (padj) < 0.05. For annotation, we used BioMart accessed through Ensembl genome browser 112.

For pathway enrichment analysis, we applied Gene Set Enrichment Analysis (GSEA) [19] using the Mouse MSigDB v2024.1.Mm database [20], ToppGene online bioinformatic platform, and Enrichr tool with BioPlanet database [21–23]. In GSEA, the entire transcriptomic dataset was used to generate a pre-ranked gene list with the scoring metric of –log10(p-value) * (logFC); all pathways with an adjusted p-value less than 0.05 were considered significantly enriched using the Benjamini-Hochberg method. For ToppGene and Enrichr functional enrichment analysis we used genes identified as significantly up- and downregulated in differential expression tests, to find the most relevant pathways, biological processes and molecular functions impacted within different data sources. Cell-type enrichment analysis was performed using the Enrichr platform to assess the transcriptional identity and potential cellular heterogeneity of Ly6G⁺ sorted populations. Differentially expressed genes were analysed against the Tabula Muris cell-type signature database.

### Data visualization and statistical analysis

Data were visualized using GraphPad Prism version 7.0.5 (GraphPad Software Inc, USA) together with RStudio by use of the ggplot2, and circlize packages [24]. Graphic figures were created using BioRender to enhance clarity and visual coherence. Statistical analysis was performed using GraphPad Prism 7.0.5. For two-group comparisons, we used two-tailed t-tests (unpaired for independent samples and paired for repeated measures) applying Welch’s correction when variances were unequal. For >2 groups, one-way ANOVA with Tukey’s post hoc test was used. When two factors were analysed simultaneously, a two-way ANOVA with an interaction term was applied, followed by appropriate post hoc tests. Associations between key echocardiographic variables (e.g., SV vs. EF, ESV vs. EF, SV vs. FS) were evaluated using Pearson’s correlation (reporting r and p) after confirming approximate normality and linearity. Data are reported as mean ± SD; all tests were two-tailed, and *p* < 0.05 was considered significant (*p < 0.05; **p < 0.01; ***p < 0.005; ***p < 0.001).

## Results

### Defining distinct phenotypes in neutrophil-like cells derived from HL-60 promyeloblasts

**Fig. 1:**
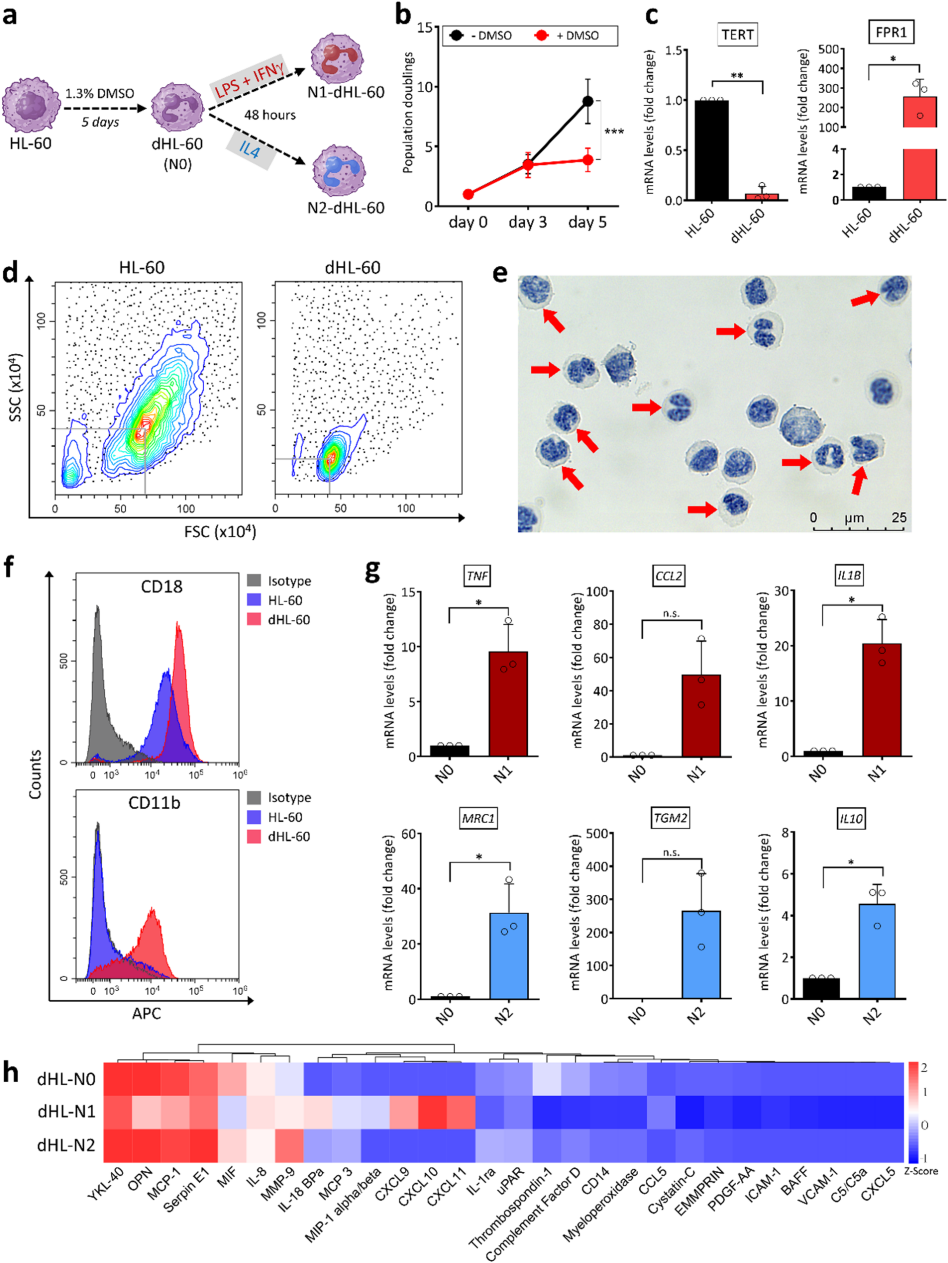
Differentiation and polarization of HL-60 cells towards pro-inflammatory (N1) and anti-inflammatory (N2) phenotypes. (a) Schematic representation of HL-60 differentiation and polarization procedures. HL-60 cells were cultured in the presence of 1.3% DMSO for 5 days to generate dHL-60 cells (N0). Subsequent stimulation with LPS + IFNγ or IL-4 for 48 h generated pro-inflammatory N1-dHL-60 and anti-inflammatory N2-dHL-60 cells, respectively. (b) Population doubling analysis of HL-60 and dHL-60 cells, showing reduced proliferation upon differentiation. Data are shown as mean ± SD; *** *p* < 0.0005. (c) Real-time RT-PCR analysis of *TERT* and *FPR1* mRNA levels in HL-60 and dHL-60 cells. dHL-60 cells exhibit a significant downregulation of *TERT* and upregulation of *FPR1* relative to HL-60 cells (data are shown as mean ± SD; *p < 0.05, **p < 0.01). (d) Flow cytometry of forward scatter (FSC) and side scatter (SSC) profiles shows the size and granularity of dHL-60 cells compared to HL-60 cells. (e) Representative image of Giemsa-stained dHL-60 cells highlighting neutrophilic morphology (red arrows). Scale bar, 25 μm. (f) Flow cytometric analysis of neutrophilic markers CD18 and CD11b expression in HL-60 and dHL-60 cells, demonstrating increased expression in dHL-60 cells. (g) Expression of pro-inflammatory (*TNF*, *CCL2*, *IL1B*) and anti-inflammatory (*MRC1*, *TGM2*, *IL10*) gene markers in dHL-60 cells polarized to N1 or N2 states. Fold changes were normalized to N0. Data are shown as mean ± SD; *p < 0.05, n.s.: not significant. (h) Heatmap of proteomic profiles secreted by dHL-60 cells under N0, N1, and N2 conditions. Hierarchical clustering of key cytokines based on Z-scores of cytokines highlights distinct transcriptional landscapes of N1- and N2-dHL-60 cells

To explore the mechanisms of neutrophil polarization and the potential influence of MSCs, we employed an in vitro system using HL-60 promyeloblast cells. These cells were differentiated into neutrophil-like cells (dHL-60) over five days with a low-dose DMSO protocol (Fig. 1a), providing a practical alternative to primary neutrophils, which have a limited lifespan in vitro. Differentiation was confirmed by significantly reduced proliferation rates in DMSO-treated cells compared to controls, as evidenced by population doublings (Fig. 1b). These changes correlated with a reduction in telomerase reverse transcriptase (*TERT*) expression and upregulation of Formyl Peptide Receptor 1 (*FPR1*), a late-stage marker indicative of neutrophil differentiation (Fig. 1c), in line with prior findings [25, 26].

Morphological analysis of the cells demonstrated a reduced cell size and a characteristic nuclear lobulation in dHL-60 cells (Fig. 1d, e), hallmark features of mature neutrophils [27]. Flow cytometry confirmed that while dHL-60 cells retained CD18 expression, they upregulated CD11b, a differentiation marker absent in HL-60 control cells (Fig. 1f). Collectively, these findings validated DMSO-treated HL-60 cells as a reliable tool for neutrophil research.

To induce polarization, dHL-60 cells were exposed for 48 h to pro-inflammatory (LPS + IFNγ) or anti-inflammatory (IL-4) conditions, adapting a protocol previously established for THP-1-derived macrophages [28, 29]. Real-time RT-PCR analysis showed a significant upregulation of *TNF*, *CCL2*, and *IL1B* gene expression in N1-dHL-60 cells, while N2-dHL-60 cells demonstrated increased expressions of *MRC1*, *TGM2*, and *IL10*, relative to unstimulated N0 controls (Fig. 1g). Further analysis of cytokine profiles revealed that N1-dHL-60 cells secreted high levels of pro-inflammatory cytokines, including CCL5, MIP-1α/β, MCP3, IL-18BPa, CXCL9, CXCL10, and CXCL11. By contrast, cytokine signature in N2-dHL-60 cells more closely resembled that of unstimulated N0 cells (Fig. 1h, Suppl. Figure 1). Together, these findings highlight that dHL-60 cells can be effectively polarized into distinct neutrophil phenotypes, paving the way for in-depth functional studies.

### Human MSCs modulate neutrophil polarization and functions through paracrine mechanisms

Building on the well-established role of MSCs in modulating macrophage polarization [30–33], we first confirmed the immunosuppressive properties of MSCs using the THP-1-derived macrophage model. THP-1 cells were differentiated into M0 macrophages using PMA, then polarized into either pro-inflammatory M1 or reparatory M2 macrophages with LPS + IFNγ or IL-4, respectively (Suppl Fig. 2a). Consistent with prior studies [34, 35], coculture with MSCs during polarization shifted M0 macrophages towards an M1 phenotype, attenuated the activation of M1 macrophages, and amplified M2 reparatory responses (Suppl Fig. 2b). These findings provided a framework for examining how MSCs influence neutrophil plasticity, a less explored aspect of MSC-mediated immune modulation.

**Fig. 2:**
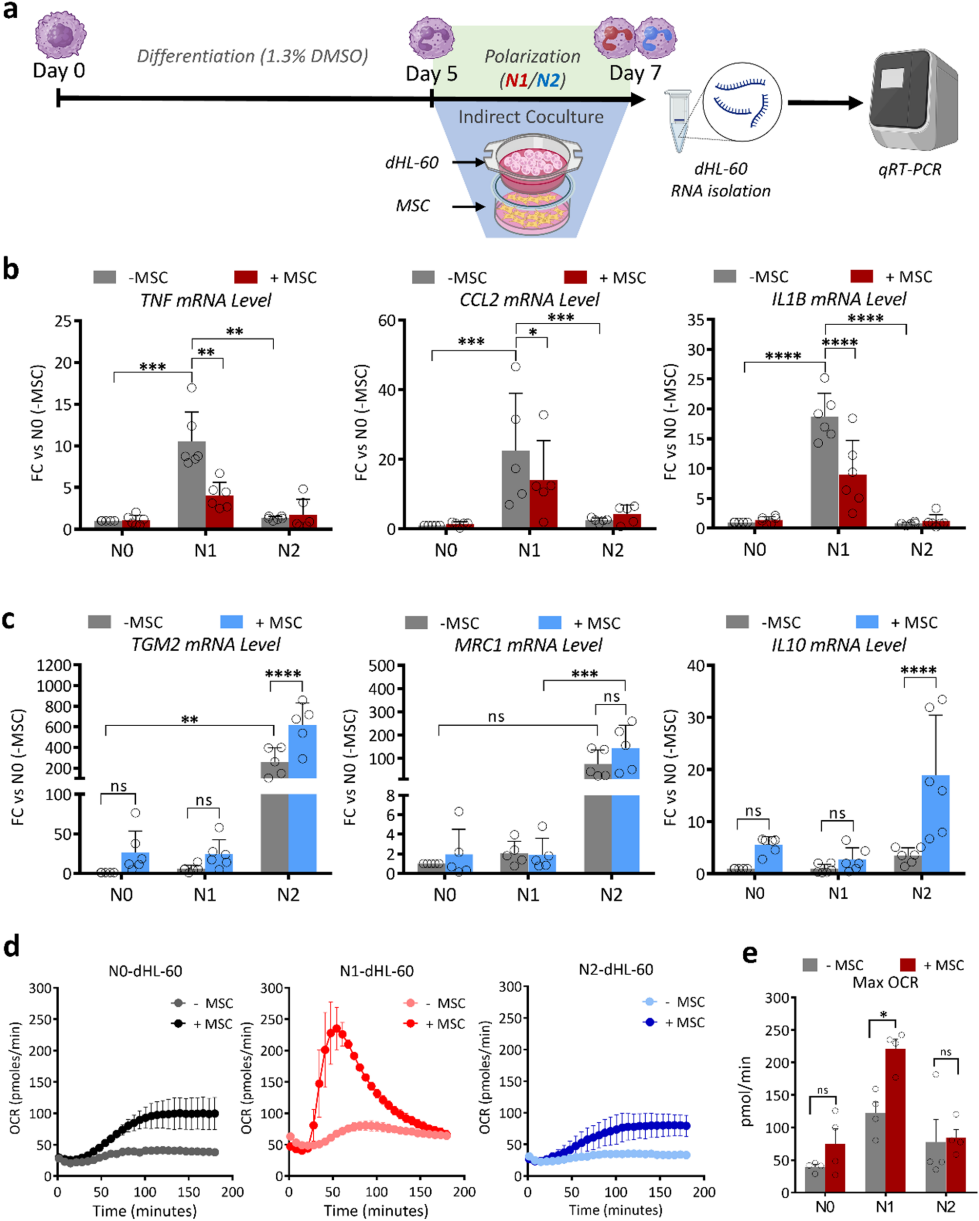
Modulation of dHL-60 polarization through indirect coculture with human MSC. **a** Schematic of the experimental workflow. Following differentiation, dHL-60 cells were indirectly cocultured with MSCs for 48 h, during their polarization to N1 (pro-inflammatory) or N2 (anti-inflammatory), followed by RNA isolation and RT-qPCR analysis. **b** RT-qPCR analysis of pro-inflammatory markers (*TNF*, *CCL2*, and *IL1B*) in dHL-60 cells (N0, N1, N2) with or without coculture with MSC. Coculture significantly reduces pro-inflammatory gene expression in N1-dHL-60 cells (Data are shown as mean ± SD; *p < 0.05, **p < 0.01, ***p < 0.001, ****p < 0.0001). **c** RT-qPCR analysis of anti-inflammatory markers (*TGM2*, *MRC1*, and *IL10*) in dHL-60 cells (N0, N1, N2) with or without coculture with MSC. Coculture enhances anti-inflammatory gene expression, particularly in N2-dHL-60 cells (Data are shown as mean ± SD; **p < 0.01, ****p < 0.0001; n.s.: not significant). **d** Oxygen consumption rate (OCR) profiles of dHL-60 cells in N0, N1, and N2 states, with or without MSC coculture. Coculture significantly elevates metabolic activity in N1-dHL-60 cells. **e** Quantification of maximum OCR in dHL-60 cells (N0, N1, N2) with or without MSC coculture. (Data are shown as mean ± SD; *p < 0.05; n.s.: not significant)

We next extended our investigations to neutrophils and employed a transwell indirect coculture model to examine the paracrine effects of MSCs. HL-60-derived neutrophils were polarized toward either the N1 or N2 phenotypes in the upper chamber, while MSCs were seeded in the lower chamber, allowing for indirect interaction over 48 h. Gene expression profiles were then analysed via RT-qPCR (Fig. 2a). Firstly, MSCs cultured under polarizing conditions showed no morphological alterations but displayed a slightly reduced proliferation rate, with no impact on viability (Suppl. Figure 3a). Notably, MSCs exposure downregulated pro-inflammatory genes (*TNF*, *CCL2*, and *IL1B*) in N1-polarized neutrophils while significantly upregulating reparatory markers (*TGM2*, *MRC1*, and *IL10*) in N2-polarized cells (Fig. 2b). These data highlight the ability of MSCs to orchestrate neutrophil polarization through secreted factors.

Functionally, we observed that phagocytic activity, measured by the cellular uptake of pHrodo-S-aureus particles, was reduced in N1-dHL-60 cells compared to N0 and N2 cells, with no impact produced by MSC coculture on phagocytic activity across all phenotypes (Suppl. Figure 3b, c). Conversely, Seahorse XF technology demonstrated an increase in the oxidative burst in MSC-cocultured neutrophils, with the most significant effect observed in N1-dHL-60 cells (Fig. 2d, e).

Overall, these findings revealed the capacity of human MSCs to modulate neutrophil phenotypes and functions through paracrine mechanisms, emphasizing their role in reprogramming neutrophil behaviour.

**Fig. 3:**
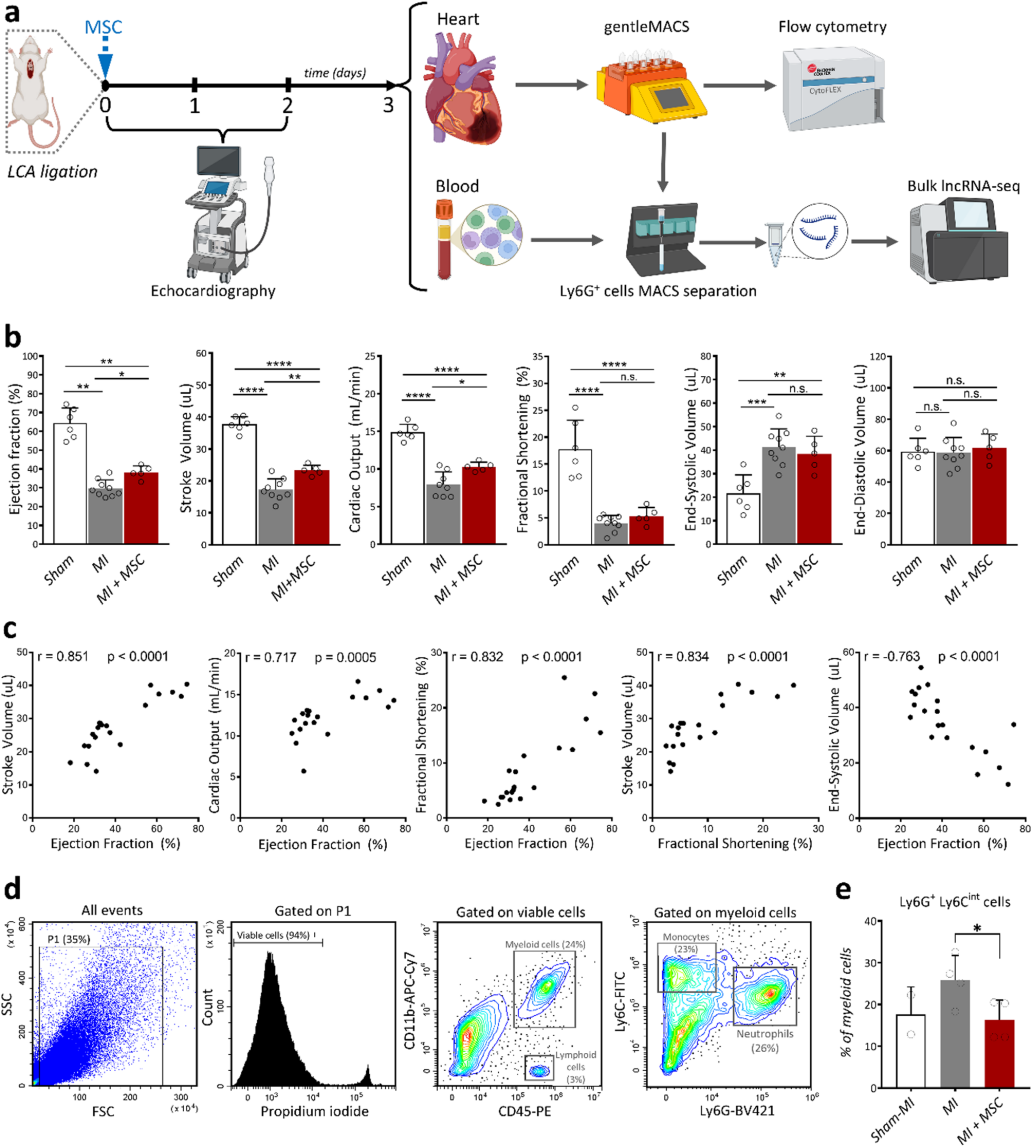
Isolation and characterization of neutrophils infiltrated following MSCs administration post-myocardial infarction. **a** Study design. Mice underwent LCA ligation (day 0) followed within 30–60 min by subcutaneous MSC injection at three different sites. Cardiac function was assessed before (sham) and 48 h post-MI by transthoracic echocardiography. Hearts and blood were harvested at day 3. Hearts were processed using gentleMACS Octo Dissociator, and cardiac cell suspension was analysed via flow cytometry (*n* = 2–4 mice/group). Ly6G⁺ cells from the heart and blood were enriched by MACS and subjected to bulk lncRNA-seq (*n* = 3 mice/group). **b** Echocardiography at 48 h post-MI (PSLAX). Bars show EF, SV, CO, FS, ESV, and EDV derived from PSLAX endocardial tracings for Sham, MI, and MI + MSC. Dots = individual mice; bars = mean ± SD. One-way ANOVA with Tukey’s post hoc; *p* < 0.05 (), < 0.01 (), < 0.001 (), < 0.0001 (****); n.s., not significant. **c** Physiological consistency of PSLAX indices. Scatterplots (all animals) display Pearson correlations between key parameters (e.g., SV vs. EF, ESV vs. EF, SV vs. FS). Expected positive associations for SV–EF/FS and a negative association for ESV–EF are observed; r and p values are shown on each panel. **d** Flow cytometric gating strategy for Ly6G⁺ myeloid cell subsets. From all events, P1 gates viable cells based on propidium iodide exclusion (left). Among viable cells, myeloid cells were identified using CD45 and CD11b expression (middle). Myeloid cells were further analyzed for Ly6G and Ly6C expression, distinguishing neutrophils (Ly6G⁺Ly6C^low/int^; right) and monocytes (Ly6G^−^Ly6C⁺; left). **e** Bar graph quantifies the percentage of Ly6G⁺ cells within the myeloid population under sham, MI-only, and MI + MSC conditions (*n* = 2–4 mice/group). Data represent mean ± SD (*p < 0.05)

### In vivo evidence showing the ability of mouse MSCs to remotely regulate neutrophil infiltration after MI

The results described above showed that MSCs are able to influence neutrophil polarization in vitro through paracrine signalling. To assess whether species-compatible paracrine effects occur in vivo, and to avoid xenogeneic confounders, a mouse MI model was employed, wherein 1 × 10^6^ syngeneic mouse MSCs were administered subcutaneously at three separate sites immediately after LCA ligation. At 48 h post-MI, subcutaneous MSC delivery was associated with an early, coherent improvement in pump performance (EF, SV, and CO significantly increased) versus MI controls, findings internally validated by strong SV-EF, FS-EF and inverse ESV-EF correlations in PSLAX-derived measurements (Fig. 3a-c). On the basis of these early functional effects, and consistent with a paracrine hemodynamic influence rather than structural remodelling, we next investigated neutrophil dynamics at day 3 post-MI, a time-point selected to capture sufficient neutrophil infiltration and the coexistence of N1/N2 subsets [36]. Mice were euthanized, and the infarcted LV was enzymatically dissociated into a single-cell suspension using a gentleMACS Octo Dissociator, and followed by flow cytometry analysis (Fig. 3a). Cardiac neutrophils were defined as CD45^+^ CD11b^+^ Ly-6C^low/int^ Ly-6G^+^ cells (Fig. 3d). Flow cytometry quantification revealed that MSC-treated mice had fewer Ly6G⁺ neutrophils in the infarcted myocardium than MI-only controls (Fig. 3e), consistent with a systemic, paracrine immunoregulatory effect of MSCs in acute myocardial injury.

**Fig. 4:**
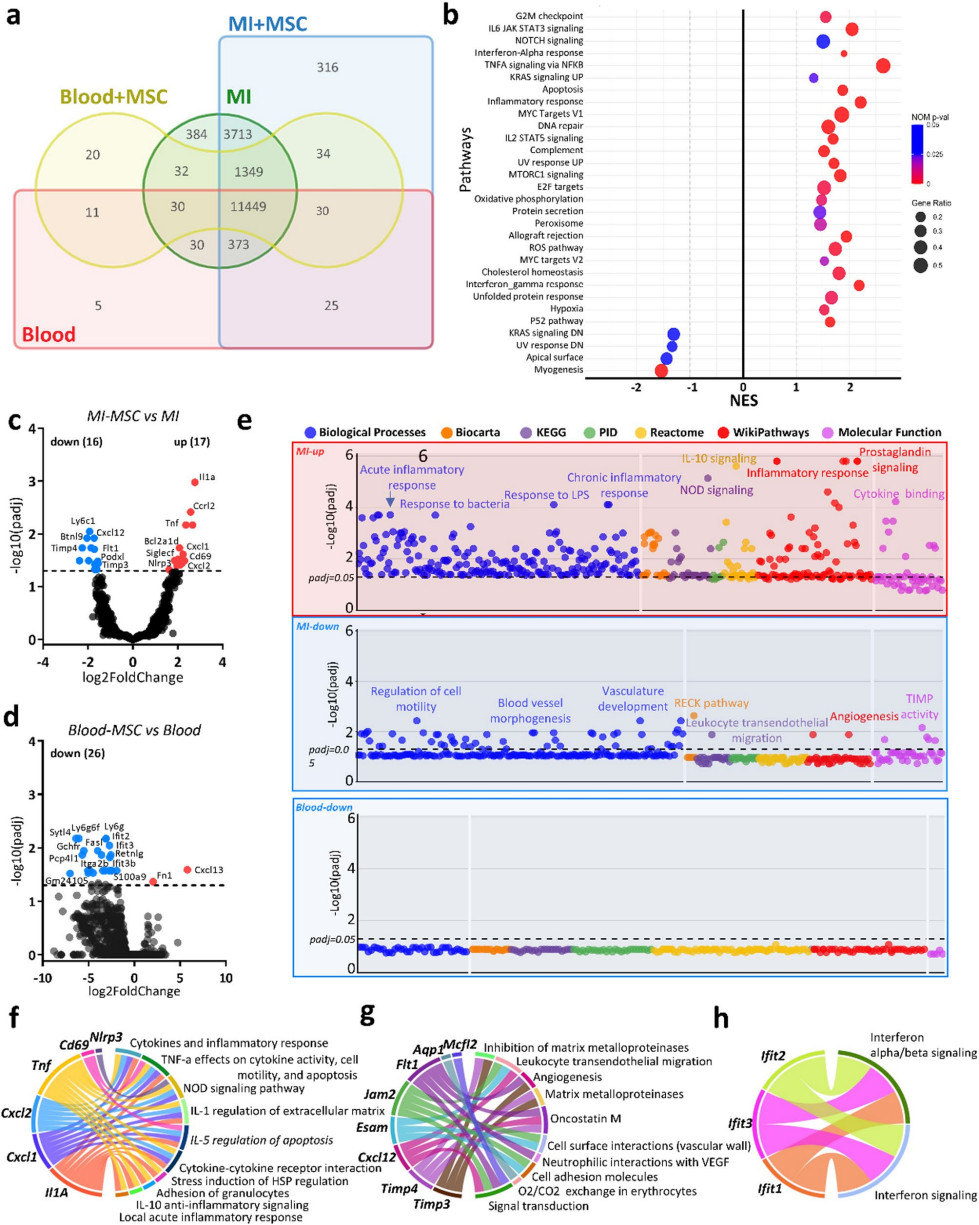
MSC-driven transcriptomic alterations in cardiac and blood neutrophils post-MI. **a** Venn Diagram illustration of coding genes among the MI, MI + MSC, Blood, and Blood + MSC groups. Normalized counts were obtained using DESeq2 and filtered for genes with mean counts ≥ 10. Numbers indicate the total coding genes unique to or shared across the groups. **b** GSEA analysis of cardiac neutrophils in MSC-treated animals vs. sham treated, based on whole-transcriptome analysis. Bubble size corresponds to the gene ratio, while colour intensity indicates p-value significance (red indicates higher significance)

**c-d** Volcano plots of differential gene expression between MI + MSC vs. MI (**c**) and Blood + MSC vs. Blood groups (**d**). Genes with significant upregulation are shown in red, downregulation in blue, and nonsignificant genes in dark gray. Dashed lines denote the significance threshold (adjusted p-value < 0.05). Gene counts for significantly regulated genes are displayed above each plot. **e** ToppGene enrichment analysis of significantly up- or downregulated genes in cardiac and blood neutrophils across pathways, biological processes, and molecular functions. The y-axis displays the − log10 adjusted p-value, while distinct colours represent various pathways or gene ontology categories, with a few representatives mentioned by name. **f-h** Chord diagrams summarizing pathway enrichment for upregulated DEG (**f**) or downregulated DEG (**g**) in cardiac and downregulated DEG (**h**) in blood neutrophils using Enrichr BioPlanet datasets. The diagrams illustrate links between enriched pathways (right) and shared genes (left), emphasizing commonalities across pathways.

### MSCs activate a pro-inflammatory phenotype in cardiac neutrophils post-MI

To investigate the molecular mechanisms driving neutrophil activation following subcutaneous MSC transplantation post-MI, we performed RNA-seq analysis on Ly6G^+^ cells isolated from both blood and infarcted cardiac tissue of mice at day 3 post-MI (Fig. 3a). Venn diagram analysis of protein-coding genes revealed a shared transcriptome of approximately 11,000 coding genes across blood and cardiac neutrophils, indicating a core expression profile in both populations. Nevertheless, neutrophils isolated from infarcted heart showed a significantly enriched transcriptome relative to blood neutrophils. Additionally, MSC-treated mice showed a higher number of uniquely expressed genes in blood neutrophils compared to sham-treated controls (Suppl. Figure 4a, Fig. 4a). To evaluate transcriptional identity and potential contamination of sorted Ly6G⁺ cells, we performed cell-type enrichment analysis using the Tabula Muris signature. Across all groups, neutrophils and granulocytes from bone marrow, fat, and lung were consistently enriched, while modest contributions from macrophage and dendritic cell signatures were also detected, suggesting a predominantly granulocytic profile with minimal heterogeneity (Suppl. Figure 4c).

In cardiac neutrophils, GSEA identified significant enrichment of inflammatory pathways including *TNFα* signalling, *IL-6/JAK/STAT3*, and interferon gamma response, as well as cell-cycle-related signatures such as G2M checkpoint, DNA repair, unfolded protein response and apoptosis (Fig. 4b, Table S2). Metabolic processes, including ROS generation and hypoxia, were also positively enriched, while pathways related to myogenesis showed negative enrichment (Fig. 4b). In contrast, blood neutrophils displayed limited transcriptional changes (Table S3), with only interferon alpha and gamma responses significantly enriched after MSC transplantation (Suppl. Figure 4b). Volcano plot analysis further underscored tissue-specific differences: in cardiac neutrophils, MSC treatment significantly altered expression of 33 genes (17 upregulated, 16 downregulated), while in blood neutrophils, only two genes (*Fn1* and *Cxcl13*) were significantly upregulated, and 26 genes were downregulated (Fig. 4c, d).

**Fig. 5:**
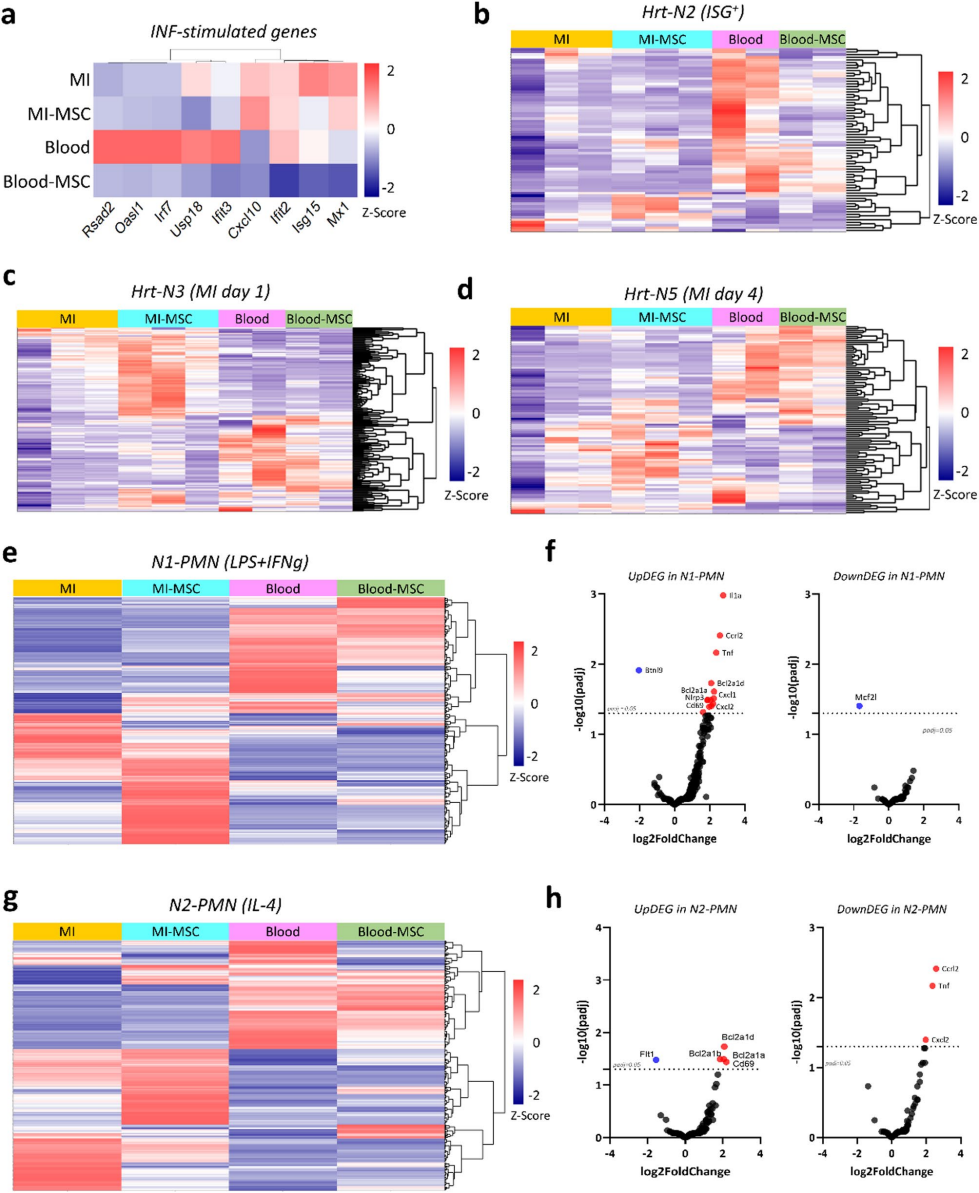
Transcriptomic profiling of neutrophil polarization by MSC remote transplantation. **a** Heatmap of interferon-stimulated genes (ISGs) across conditions: MI, MI + MSC, blood, and blood + MSC. MSC treatment modulates ISG expression in the blood, reducing inflammation-associated signatures. **b-d** Heatmap for the hierarchical clustering of differentially expressed gene profiles in neutrophil subsets as characterized by Calcagno et al.: Hrt-N2 characterized by ISG expression (**b**), Hrt-N3, identified at day 1 post-MI (**c**), and Hrt-N5, identified at day 4 post-MI (**d**), showed distinct transcriptional profiles modulated by MSC administration. **e** Heatmap of transcriptional profiles of cardiac and blood neutrophils based on N1-polarized neutrophils (N1-PMN; stimulated with LPS + IFNγ) as characterized by Mihaila et al. MSC treatment attenuates pro-inflammatory N1-PMN transcriptional profile in blood neutrophils while increasing it in cardiac neutrophils. **f** Volcano plots of DEGs from N1-PMNs, showing upregulated (red) and downregulated (blue) genes following MSC treatment in cardiac neutrophils. MSC treatment significantly impacts several key pro-inflammatory and anti-inflammatory genes (labelled). **g** Heatmap of transcriptional profiles of cardiac and blood neutrophils based on N2-polarized neutrophils (N2-PMN; stimulated with IL-4) as characterized by Mihaila et al. MSC treatment significantly altered the anti-inflammatory N2-PMN transcriptional profile in both MI-derived and blood neutrophils. **h** Volcano plots of DEGs from N2-PMNs, showing upregulation of both upregulated DEGs (left) and downregulated DEG (right) genes in MI-derived neutrophils by MSC treatment

Further pathway enrichment analysis using ToppGene and Enrichr provided additional insights into the specific biological states including biological processes and molecular functions. To this, we queried only the significant upregulated or downregulated DEGs against curated pathway databases such as Gene Ontology (GO), Biocarta, KEGG, Reactome and WikiPathways (for ToppGene) or Bioplanet (for Enrichr). ToppGene analysis revealed that, in cardiac neutrophils of MSC-treated animals, upregulated DEGs were primarily associated with immune activation and inflammatory responses, while downregulated DEGs were linked to angiogenesis, vascular remodelling, and leukocyte migration. Blood neutrophils displayed no significant pathway enrichment among downregulated DEGs (Fig. 4e). Enrichr analysis identified clusters of upregulated genes (*Nlrp3*,* Cd69*,* Tnf*,* Cxcl2*,* Cxcl1*, and *Il1a*) linked to an enriched inflammatory pathway, while downregulated genes (*Timp3*,* Timp4*,* Cxcl12*,* Esam*,* Jam2*,* Flt1*,* Aqp1* and *Mcfl2*) were associated with tissue remodelling, angiogenesis, and immune cell migration (Fig. 4f, g). In blood neutrophils, MSCs suppressed a small cluster of interferon-response genes (*Ifit1*,* Ifit2*,* Ifit3*), which are involved in adverse cardiovascular outcomes (Fig. 4h) [37].

Overall, these findings highlight that MSCs selectively modulate neutrophil phenotypes and functions, in a tissue-specific manner, promoting inflammatory pathways in the cardiac microenvironment.

### Validation of MSC-induced transcriptomic changes in neutrophils using independent datasets

To validate our findings, we compared MSC-induced transcriptomic changes in neutrophils with published RNA-seq datasets [8, 38]. Calcagno et al. previously reported that subsets of circulating neutrophils and monocytes express interferon-stimulated genes (ISG) before infiltrating the infarcted heart, suggesting that innate immune pathways could be remotely activated [8]. We investigated whether the enrichment of interferon signalling identified in blood neutrophils from MSC-treated mice could be assessed by the ISG score identified by Calcagno et al. Our analysis revealed that MSC transplantation significantly reduced the ISG signature in circulating neutrophils but had no impact on cardiac neutrophils (Fig. 5a-b). To further explore MSC-induced transcriptional changes, we aligned our data with the five intracardiac neutrophil subsets defined by Calcagno et al. Notably, MSC treatment enhanced the neutrophil subset associated with NF-κB activation in day 1 post-MI (Fig. 5c). In contrast, the subset linked to reparative neutrophils at later stages of MI (day 4), remained unaffected in both blood and cardiac neutrophils (Fig. 5d).

To extend these insights, we examined how MSC-induced transcriptional changes aligned with previously characterized N1 and N2 neutrophil transcriptomes [38], in order to determine if remote MSC transplantation could transcriptomically mirrors the N1/N2 phenotypes in blood or MI neutrophils. Heatmap analysis revealed pronounced transcriptional changes in cardiac neutrophils from MSC-treated animals, while blood neutrophils showed only minor changes (Fig. 5e, g). Upregulated genes associated with N1 neutrophils were significantly increased in cardiac neutrophils, suggesting a pro-inflammatory influence of MSCs within the infarcted heart (Fig. 5f). However, blood neutrophils showed a mixed N1/N2 pattern, indicating a less defined impact on the reparatory phenotype (Fig. 5h).

### Independent verification of transcriptomic changes in MSC-modulated neutrophils after MI

**Fig. 6:**
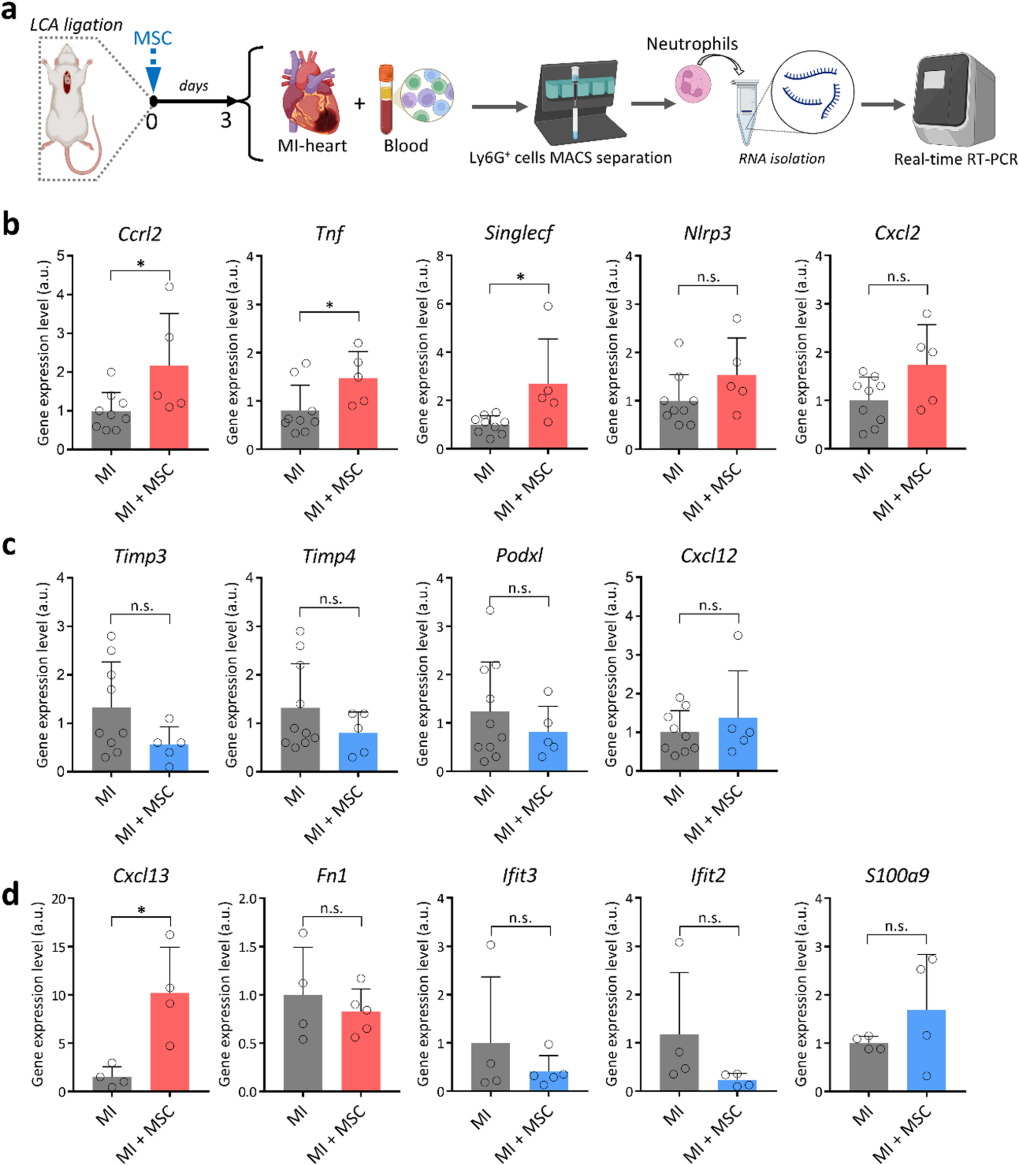
MSC-associated transcriptional changes in murine neutrophils 3 days after MI. **a** Experimental timeline: LCA ligation was performed on day 0, followed by subcutaneous MSC administration. On day 3, hearts and blood were collected, Ly6G⁺ cells were enriched by MACS, and RNA was extracted for RT-qPCR analysis. **b-d** RT–qPCR in Ly6G⁺ neutrophils from the infarcted heart **b**,** c** and peripheral blood **d** of MI and MI + MSC mice. Panel **b** shows genes identified as up-regulated by RNA-seq in cardiac neutrophils (*Ccrl2*, *Tnf*, *Siglecf*, *Nlrp3*, *Cxcl2*); panel **c** shows down-regulated genes (*Timp3*, *Timp4*, *Podxl*, *Cxcl12*); panel **d** shows selected transcripts profiled in blood neutrophils (*Cxcl13*, *Fn1*, *Ifit3*, *Ifit2*, *S100a9*). Data are mean ± SD with individual mice shown (*n* = 4–9 per group). Expression values were normalized to housekeeping genes and plotted as arbitrary units (a.u.). Statistics: unpaired two-tailed t-test (MI vs. MI + MSC); *p* < 0.05 (*); n.s., not significant

To orthogonally validate the transcriptomic signatures, we quantified selected transcripts by RT–qPCR in Ly6G⁺ neutrophils purified from the infarcted left ventricle and peripheral blood of an expanded MI cohort at day 3 (Fig. 6a). In cardiac neutrophils, MSC treatment significantly increased *Ccrl2*, *Tnf*, and *Siglecf*, with concordant upward trends for *Nlrp3* and *Cxcl2* (Fig. 6b), while repair/retention genes (*Timp3*, *Timp4*, *Podxl*, *Cxcl12*) showed no significant change (Fig. 6c). Circulating neutrophils exhibited the same directional shifts predicted by RNA-seq (*Cxcl13*, *Fn1*, *Ifit3*, *Ifit2*, *S100a9*), although most single-gene changes were not statistically significant (Fig. 6d). Overall, these targeted gene-expression measurements corroborate the directionality of key RNA-seq findings and indicate that MSCs mainly modulate inflammatory gene programs in cardiac, rather than blood, neutrophils at this early time point post-MI.

### Translational validation of MSC–neutrophil paracrine crosstalk

**Fig. 7:**
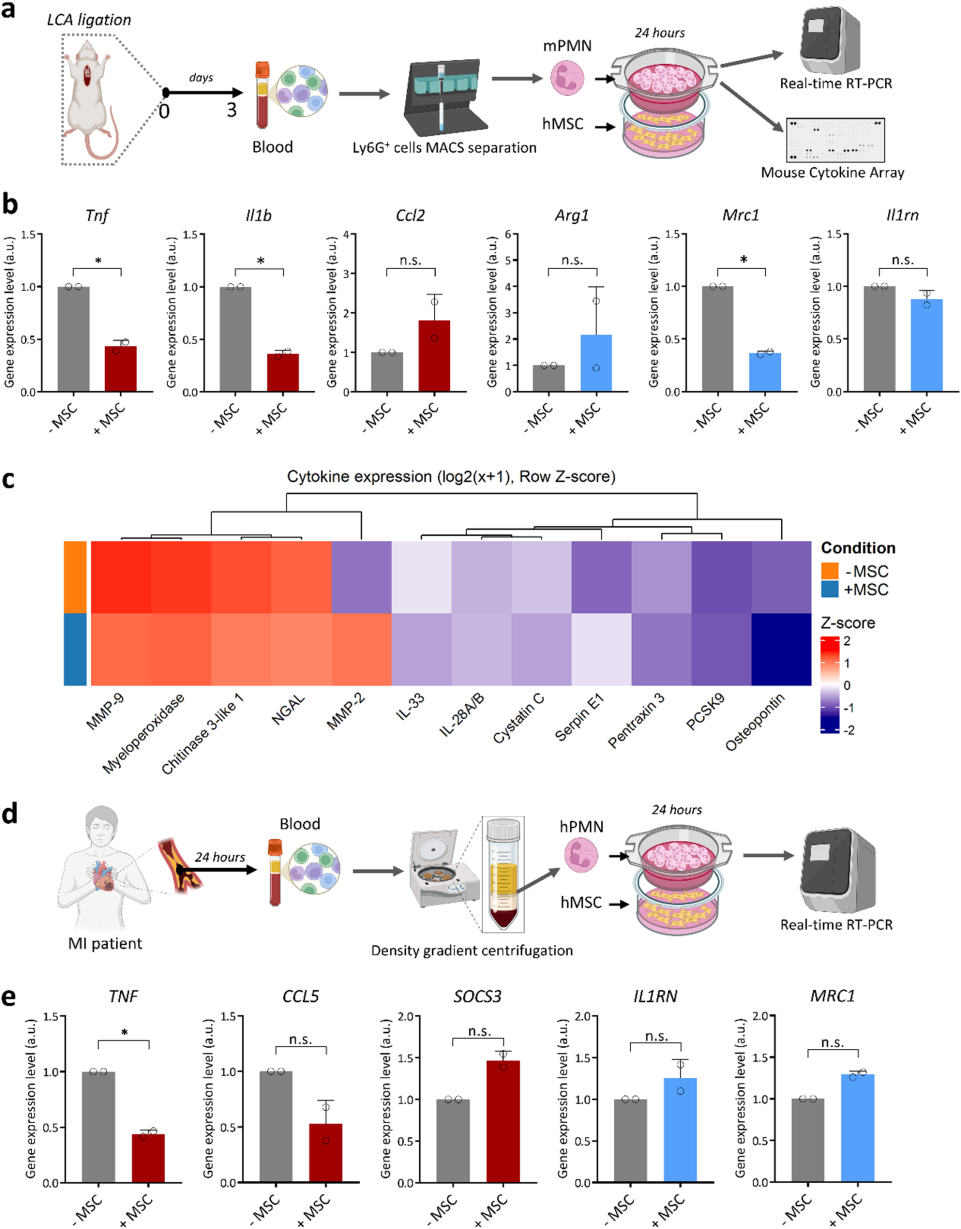
Paracrine crosstalk with MSCs modulates inflammatory outputs of circulating mouse and human neutrophils post-MI. **a** Mouse ex vivo workflow. At day 3 after LCA ligation, peripheral blood was collected and Ly6G⁺ neutrophils were purified by MACS and cultured for 24 h in 0.4-µm transwell inserts above human MSC monolayers (indirect coculture, ±MSC). Neutrophils were then harvested for RT–qPCR, and conditioned media were assayed with a Mouse XL Cytokine Array. **b** Expression of selected inflammatory and reparatory genes (*Tnf*,* Il1b*,* Ccl2*,* Arg1*,* Mrc1*,* Il1rn*) in blood neutrophils cultured alone (–MSC) or with human MSCs (+ MSC). Dots represent independent experiments (*n* = 2); bars show mean ± SD. Expression was normalized to housekeeping genes and plotted as arbitrary units (a.u.). Statistics: paired two-tailed t-test; *p* < 0.05 (*); n.s., not significant. **c** Mouse cytokine array. Heat map of secreted proteins (log₂[x + 1], row Z-score) measured in supernatants from mouse neutrophils cultured alone (–MSC) or with human MSCs (+ MSC), highlighting changes across targets, with the biggest difference identified in the expression level of MMP-2. **d** Human ex vivo workflow. Peripheral blood was obtained from MI patients within 24 h of presentation (ethics/consent obtained). Human neutrophils (hPMN; MACS-enriched) were cocultured 24 h with human MSCs in transwells (± MSC) and analysed by RT–qPCR. **e** Transcript levels of *TNF*, *CCL5*, *SOCS3*, *IL1RN*, and *MRC1* in human neutrophils cultured alone (–MSC) or with human MSCs (+ MSC) for 24 h. Dots denote independent experiments (*n* = 2); bars show mean ± SD. Normalization and statistics as in panel b

Finally, to evaluate the translational relevance of MSC–neutrophil paracrine crosstalk, we performed short-term indirect ex vivo cocultures with primary post-MI neutrophils. First, murine blood Ly6G⁺ neutrophils harvested at day 3 post-MI were cocultured with human MSCs monolayers for 24 h (Fig. 7a). RT-qPCR analysis showed significant reductions in the proinflammatory transcripts *Tnf* and *Il1b*, accompanied by a decrease in the anti-inflammatory marker *Mrc1* (Fig. 7b). Secretome profiling demonstrated an attenuation of degranulation/remodelling factors (e.g., MMP-9, MPO, NGAL) in the presence of MSCs, while MMP-2 was significantly increased (Fig. 7c, Suppl. Figure 1). Importantly, blood neutrophils from patients with acute MI cocultured with human MSCs for 24 h exhibited a significant reduction in *TNF* transcript, with *CCL5*, *SOCS3*, *IL1RN* and *MRC1* showing concordant but non-significant trends (Fig. 7d, e). These data indicate that the *TNF*-suppressive effect is conserved across species, but a comprehensive shift to an N2-like state is not evident over 24 h.

Collectively, these findings reveal previously unrecognized effects of MSCs on neutrophil transcriptomes within the MI settings, with potential implications for the post-MI inflammation and tissue remodelling.

## Discussion

Following myocardial infarction, the heart undergoes an inflammatory cascade that consists of an initial pro-inflammatory phase followed by the clearance of cellular debris, through a resolution phase that activates the reparative program [39]. This progression involves neutrophils as early responders, followed by monocytes and macrophages that orchestrate tissue repair. Neutrophils clear necrotic tissue and release mediators that activate macrophages and monocytes for tissue repair. This response is characterized by neutrophil polarization from an N1 inflammatory state to an N2 reparative state, a shift essential for resolution and healing [39]. However, these processes culminate in fibrotic scar formation, which hinders cardiac recovery [6].

MSCs are recognized for their immunomodulatory properties in post-MI settings, notably through macrophage polarization toward reparative M2 phenotypes [13, 14, 40]. This study investigated the underexplored role of MSCs in neutrophil polarization, revealing three important findings: (i) MSCs promote an anti-inflammatory N2-like neutrophil phenotype in vitro via paracrine signalling; (ii) remote transplantation of MSCs reduces neutrophil infiltration into infarcted myocardium and improves early cardiac performance, (iii) MSCs induce significant transcriptomic changes in neutrophils, predominantly affecting inflammatory signalling pathways in the MI microenvironment.

Functional and transcriptomic plasticity of neutrophils has been documented in cancer and MI, where polarization into N1 and N2 states has been validated in vitro and in vivo [36, 38, 41]. Using HL-60 cells differentiated into neutrophil-like cells, which circumvents the limited lifespan of primary neutrophils in vitro [42–44], we established an in vitro model to study N1/N2 polarization, and their responsiveness to biomolecules secreted by MSCs. We demonstrated that MSCs significantly downregulated pro-inflammatory genes in N1-dHL-60 cells while upregulating N2 markers such as TGM2 and IL-10. These findings are consistent with earlier reports of MSC-mediated neutrophil polarization through paracrine mechanisms [45, 46]. Moreover, we observed a metabolic shift in neutrophils cocultured with MSCs, from the primarily glycolytic metabolism of these cells to an enhanced oxidative metabolism. This change boosts the ability of the cells to produce ROS and therefore perform the characteristic oxidative burst [47, 48].

Nevertheless, understanding the impact of MSCs on inflammatory responses in vivo is essential for advancing MSC-based therapies. Subcutaneous transplantation of MSC, a minimally invasive delivery method, has shown promise in reducing systemic inflammation and protecting distant organs without requiring direct migration to the injury site [11]. Neutrophils, which play a critical role in the inflammatory phase, being increasingly recognized as contributors to the reparative phase [36, 49], are key targets for MSC-mediated immunomodulation. It was shown that MSCs promoted neutrophil apoptosis, which subsequently enhanced macrophage polarization toward reparative phenotypes, thereby reducing inflammation and supporting cardiac repair [8, 50].

Elevated neutrophil counts are associated with poor clinical outcomes and increased mortality in patients with acute coronary syndromes [51, 52]. However, while post-MI neutrophil depletion promotes macrophage polarization toward reparative phenotypes, it also worsens cardiac function, increases fibrosis, and leads to progressive heart failure in murine models [53]. Yet, neutrophil reduction may significantly influence the transition between the inflammatory and reparative phases during myocardial healing [54]. Our data demonstrate that MSC remote transplantation has an early effect on cardiac performance that reflects paracrine immunoregulatory actions rather than a structural remodelling of the heart. Moreover, MSCs effectively limit inflammatory leukocyte infiltration post-MI, highlighting MSCs systemic immunomodulatory capability. Previous studies reported a temporal polarization of neutrophils in the left ventricle post-MI [53, 55], while single-cell transcriptomics provided further insights into the heterogeneity of cardiac cellular responses [8, 56, 57].

The molecular mechanisms underlying neutrophil-MSC crosstalk in vivo remain poorly understood. While MSCs are widely recognized for their anti-inflammatory properties [12], our work uniquely demonstrates that remote MSC transplantation is associated with transcriptomic enrichment of pro-inflammatory programs in cardiac neutrophils, suggesting a compartment-specific response to MSC therapy after MI. A prudent explanation is indirect shaping of the myocardial chemokine/cytokine milieu by MSCs, which could skew recruitment toward earlier, more inflammatory neutrophil subsets. Alternatively, MSC-derived paracrine cues may promote phenotypic plasticity of infiltrating neutrophils, re-imprinting them within the ischemic microenvironment. A third, non-exclusive mechanism is MSC-mediated modulation of other immune effectors (e.g., macrophages or T cells) that secondarily direct neutrophil activation states via cross-talk. While our findings do not resolve causality among these possibilities, they refine the prevailing view of MSCs as uniformly anti-inflammatory and highlight the need to consider systemic dampening versus local re-programming as separable axes of MSC action.

Together, our mouse and human ex vivo data independently validate the MSC–neutrophil paracrine interaction observed in vivo and support its likely translational relevance by demonstrating that neutrophils post-MI are responsive to MSC-derived signals. Particularly, secretome profiling showed an increased secretion of MMP-2 and Serpin E1 by day-3 post-MI neutrophils in the presence of MSCs, indicating also an MSC-driven shift toward ECM remodelling and controlled fibrinolysis.

It is of note that, the targeted RT-qPCR validation broadly corroborated the RNA-seq findings and underscores a compartment-specific reprogramming of neutrophils by remote MSC delivery after MI. While some qPCR comparisons were underpowered, the overall concordance in direction with RNA-seq strengthens the conclusion that MSCs differentially modulate neutrophil programs in blood versus infarcted myocardium. The convergence of in vivo function, transcriptomics, RT-qPCR, and ex vivo human data strengthens this interpretation and refines expectations for MSC therapy: MSCs temper neutrophil effector programs systemically but their net impact within the infarct is dictated by local cues.

This dichotomy challenges the general view of MSCs as uniform anti-inflammatory agents and highlights the complexity of MSC-neutrophil interactions, emphasizing the need for context-dependent analyses to fully understand MSC functions in vivo. When considered within the broader landscape of in vivo cellular interactions, these findings align with the dynamic behaviour of various cell populations [58]. They also shed light on how MSCs influence the microenvironment at sites of injury, providing a more nuanced understanding of their role in immune modulation.

### Study limitations

While our study provides significant insights into the therapeutic potential of subcutaneous MSC transplantation, some areas warrant further exploration. A main limitation of this study is the species mismatch between the experimental systems. Although HL-60 differentiation provides a reproducible platform and human MSCs are highly relevant to clinical translation, the behaviour of these human cells may not faithfully recapitulate that of primary murine MSCs and neutrophils within the post-MI cardiac microenvironment. This raises the concern that the divergent responses observed in vitro and in vivo may, at least in part, reflect a species mismatch rather than differences in the experimental setup. We partially address this by complementing the in vivo model with ex vivo cocultures of human MSCs with primary peripheral neutrophils obtained from MI mice and from MI patients, which showed directionally consistent suppression of pro-inflammatory transcripts and modest effects on selected regulatory markers. Other limitations may include the in vivo use of syngeneic mouse MSCs at a single subcutaneous dose/time point. Thus, our findings may not generalize to other MSC sources, doses, routes, or temporal windows. Finally, some ex vivo cytokine and coculture assays were performed with limited replicates, and several qPCR trends did not reach statistical significance, warranting confirmation in larger, prospectively powered experiments and with additional MSC doses/routes and time points. Collectively, these constraints temper over-generalization but do not weaken the central conclusion that MSCs can modulate neutrophil programs relevant to post-MI inflammation through paracrine mechanisms.

## Conclusions

In summary, our results indicate that anti-inflammatory properties of MSCs should not be viewed as a general mechanism, but rather as an adaptive process shaped by cell types and tissue-specific microenvironments. While the study deepens our understanding of MSC–neutrophil interactions and highlights important clues for refining MSC-based strategies to balance inflammation and repair post-MI, further studies in clinically relevant humanized systems are essential to confirm translational relevance.

## Supplementary Information

Below is the link to the electronic supplementary material.

Supplementary Material 1.

Supplementary Material 2. fig 1 Proteome profile analysis of dHL-60 secretome in unstimulated N0 state compared with N1 or N2 polarization phenotypes (top), and secretome of mouse neutrophils from MI-mice after 24 hours of indirect coculture with human MSC in vitro.

Supplementary Material 3. Fig 3 MSC-mediated modulation of macrophage polarization in vitro. (a) Schematic of the experimental design. THP-1 monocytic cells were differentiated into M0 macrophages (M0 Mac) using PMA (100 nM, 72 h). M0 macrophages were polarized toward M1 (pro-inflammatory; LPS+IFNγ, 48 h) or M2 (anti-inflammatory; IL-4, 48 h) phenotypes, either in the absence (-MSC) or presence (+MSC) of human MSCs in coculture. Marker genes for M1-like macrophages (e.g., TNF, IRF7, CCL5, SOCS3) and M2-like macrophages (e.g., MRC1, TGM2, CCL22, CCL17) were analysed by RT-qPCR. Representative phase-contrast images of THP-1 cells, M0 macrophages, and polarized M1/M2 macrophages are shown.(b) mRNA levels of M1 markers (TNF, IRF7, CCL5, SOCS3) normalized to M0 (-MSC). MSC treatment significantly reduced M1 marker expression compared to control conditions, indicating suppression of pro-inflammatory polarization.(c) mRNA levels of M2 markers (MRC1, TGM2, CCL22, CCL17) normalized to M0 (-MSC). MSC treatment enhanced the expression of these markers, supporting the promotion of anti-inflammatory and reparative macrophage polarization.

Supplementary Material 4. Fig 3: Effects of MSC-dHL-60 coculture on cell behaviour and function. a Representative phase-contrast images showing the morphological changes of MSCs under various culture conditions. MSCs were cultured either in MSC-specific growth medium (as control) or in HL-60-specific medium, in the absence (N0) or presence of the polarizing growth factors, LPS+IFNγ (typical for N1), or IL-4 (typical for N2), for 24 or 48 hours. MSC morphology was not affected, yet a modest decrease in cell proliferation was noted in HL-60 medium. Scale bar = 50 μm. b Flow cytometric analysis of dHL-60 phagocytosis activity using pHrodo-FITC. Polarized dHL-60 cells (N0, N1, N2) were cocultured with or without MSCs, and phagocytosis was quantified by the uptake of pHrodo-labelled particles. The proportion of pHrodo-positive cells is indicated in each quadrant. c Quantification of phagocytic activity. Mean fluorescence intensity (MFI) of pHrodo-FITC was used as an indicator of phagocytosis. MSC coculture do not modify the phagocytic function of N0-N1-N2 dHL-60 cells.

Supplementary Material 5. Fig 4 Transcriptomic analysis of neutrophils. a Bar plot from Venn diagram analysis displaying the overlap of differentially expressed genes (DEGs) in Ly6G⁺ neutrophils isolated at day 3 post-MI from infarcted heart or blood, in mice treated with or without MSCs. The number of unique and shared DEGs between conditions is indicated. b GSEA plot showing the enriched signalling pathways activated in blood neutrophils in response to MSC transplantation. Normalized enrichment scores (NES) are depicted, with statistical significance denoted by the p-value in gradient, and dot size represents gene ratio. c Cell-type enrichment analysis using the Tabula Muris reference signature. Plots show the top significantly enriched immune cell types (Fisher’s exact test, p-adj < 0.05) based on cell origin across the four conditions.

## Data Availability

The RNA-seq datasets described in this study have been deposited in NCBI’s Gene Expression Omnibus (GEO) and are accessible through GEO Series accession number GSE287432. All other data that support the findings on this study are available in the supplementary data files or from the corresponding author upon reasonable request.
