# Supplementary figures and images for: Mesenchymal stromal cells modulate neutrophil phenotype via paracrine signals
Source: Stem Cell Res Ther. 2025 Oct 21;16:574. doi: 10.1186/s13287-025-04684-w (PMC12539047; doi:10.1186/s13287-025-04684-w)

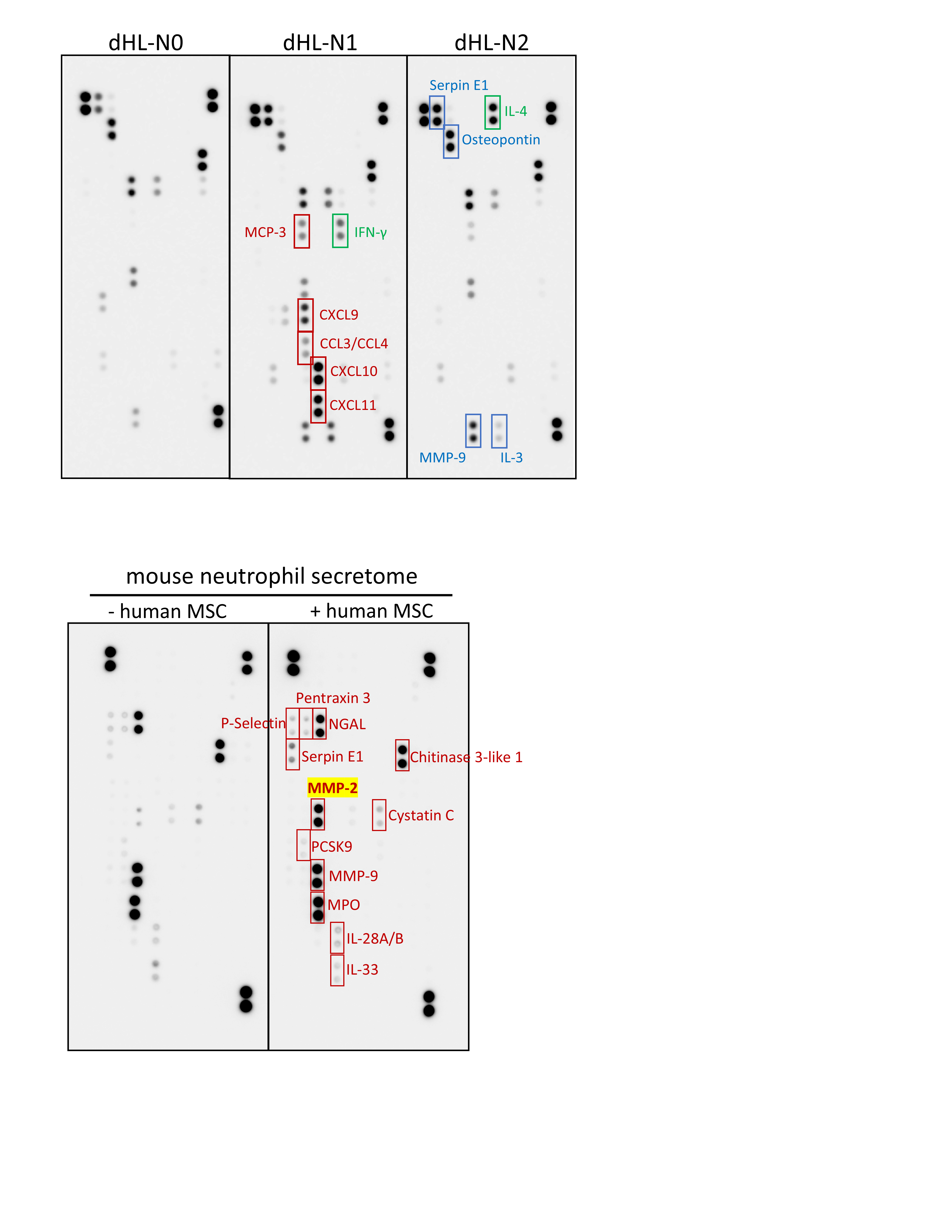

Supplement: Supplementary file 2 — Supplementary Material 2. fig 1 Proteome profile analysis of dHL-60 secretome in unstimulated N0 state compared with N1 or N2 polarization phenotypes (top), and secretome of mouse neutrophils from MI-mice after 24 hours of indirect coculture with human MSC in vitro. [file 13287_2025_4684_MOESM2_ESM.png]

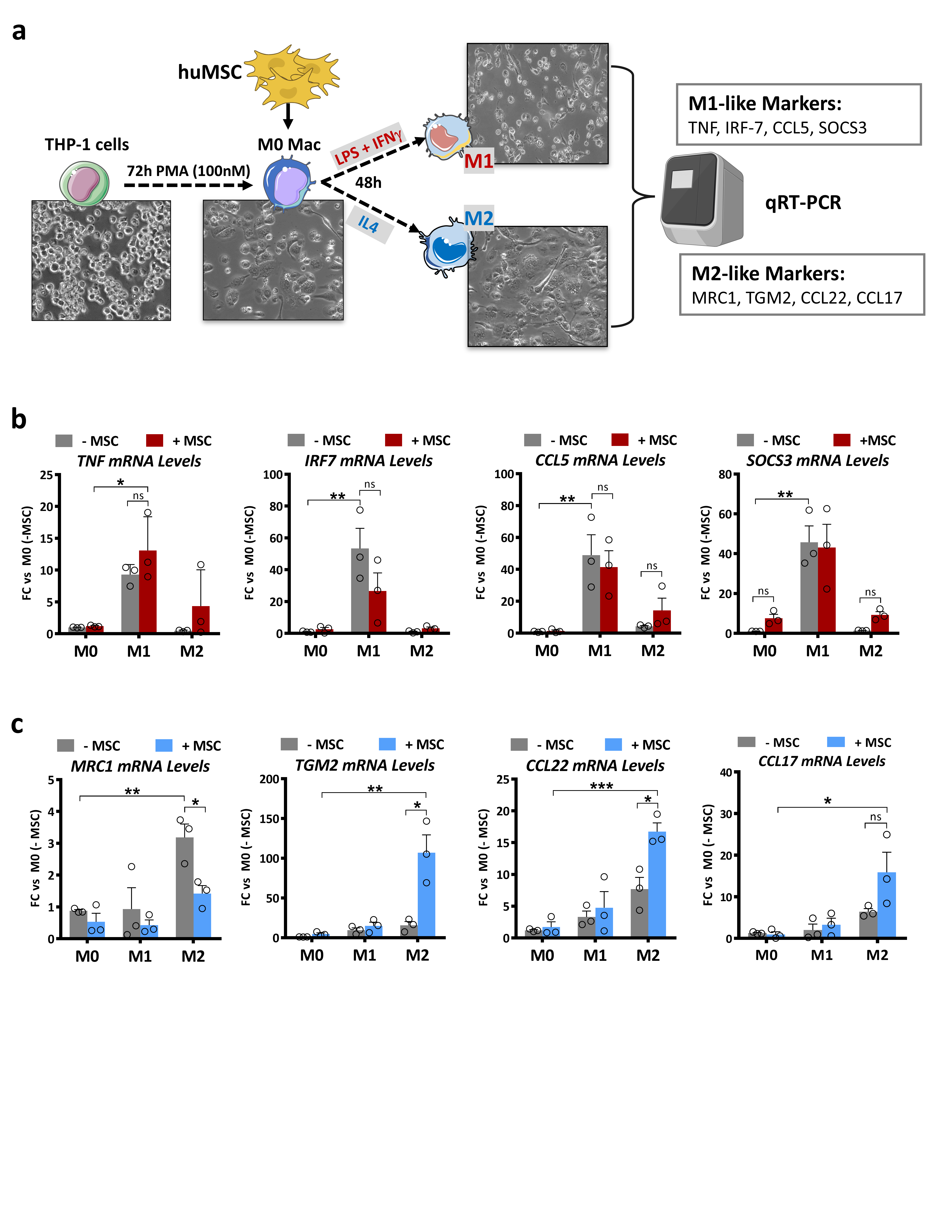

Supplement: Supplementary file 3 — Supplementary Material 3. Fig 3 MSC-mediated modulation of macrophage polarization in vitro. (a) Schematic of the experimental design. THP-1 monocytic cells were differentiated into M0 macrophages (M0 Mac) using PMA (100 nM, 72 h). M0 macrophages were polarized toward M1 (pro-inflammatory; LPS+IFNγ, 48 h) or M2 (anti-inflammatory; IL-4, 48 h) phenotypes, either in the absence (-MSC) or presence (+MSC) of human MSCs in coculture. Marker genes for M1-like macrophages (e.g., TNF, IRF7, CCL5, SOCS3) and M2-like macrophages (e.g., MRC1, TGM2, CCL22, CCL17) were analysed by RT-qPCR. Representative phase-contrast images of THP-1 cells, M0 macrophages, and polarized M1/M2 macrophages are shown.(b) mRNA levels of M1 markers (TNF, IRF7, CCL5, SOCS3) normalized to M0 (-MSC). MSC treatment significantly reduced M1 marker expression compared to control conditions, indicating suppression of pro-inflammatory polarization.(c) mRNA levels of M2 markers (MRC1, TGM2, CCL22, CCL17) normalized to M0 (-MSC). MSC treatment enhanced the expression of these markers, supporting the promotion of anti-inflammatory and reparative macrophage polarization. [file 13287_2025_4684_MOESM3_ESM.png]

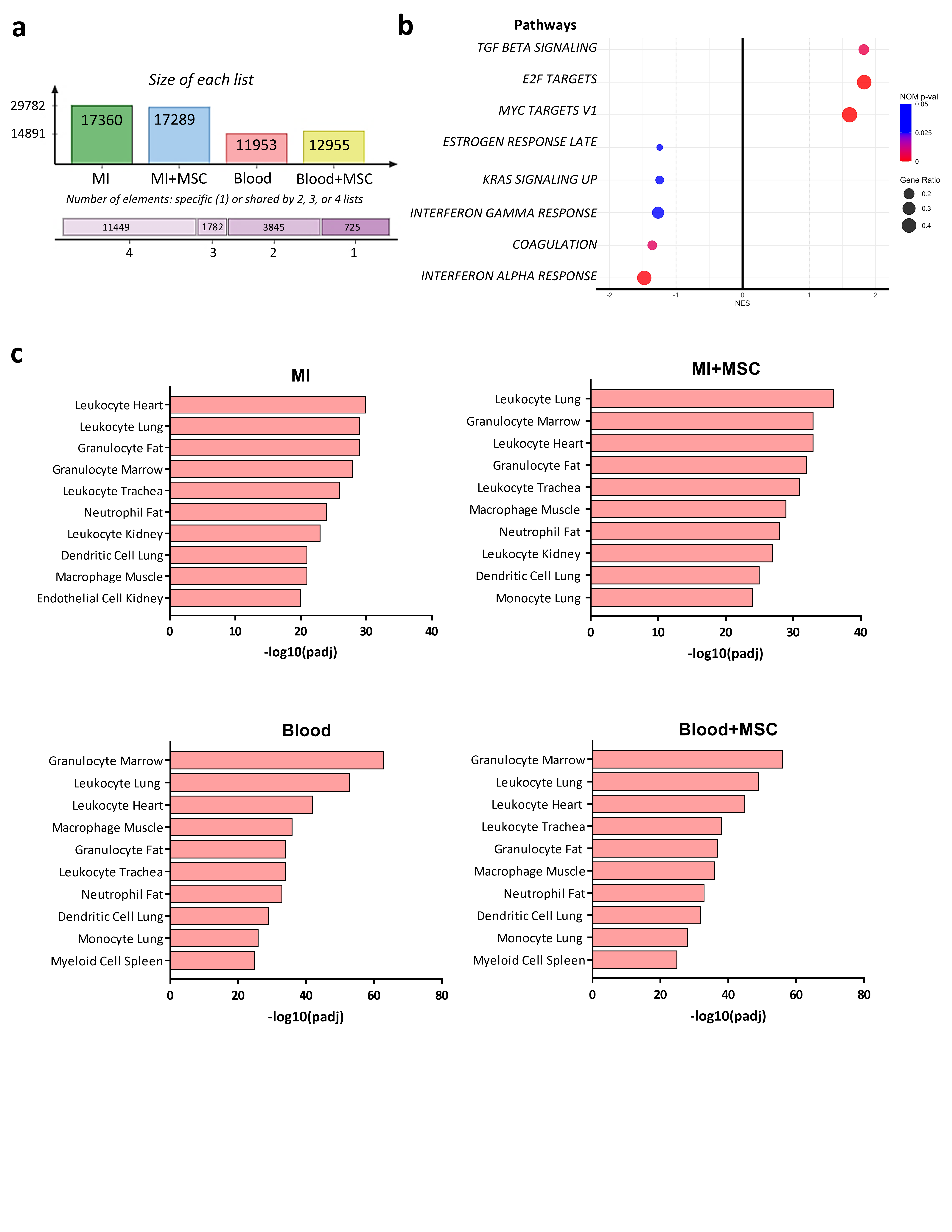

Supplement: Supplementary file 4 — Supplementary Material 4. Fig 3: Effects of MSC-dHL-60 coculture on cell behaviour and function. a Representative phase-contrast images showing the morphological changes of MSCs under various culture conditions. MSCs were cultured either in MSC-specific growth medium (as control) or in HL-60-specific medium, in the absence (N0) or presence of the polarizing growth factors, LPS+IFNγ (typical for N1), or IL-4 (typical for N2), for 24 or 48 hours. MSC morphology was not affected, yet a modest decrease in cell proliferation was noted in HL-60 medium. Scale bar = 50 μm. b Flow cytometric analysis of dHL-60 phagocytosis activity using pHrodo-FITC. Polarized dHL-60 cells (N0, N1, N2) were cocultured with or without MSCs, and phagocytosis was quantified by the uptake of pHrodo-labelled particles. The proportion of pHrodo-positive cells is indicated in each quadrant. c Quantification of phagocytic activity. Mean fluorescence intensity (MFI) of pHrodo-FITC was used as an indicator of phagocytosis. MSC coculture do not modify the phagocytic function of N0-N1-N2 dHL-60 cells. [file 13287_2025_4684_MOESM4_ESM.png]

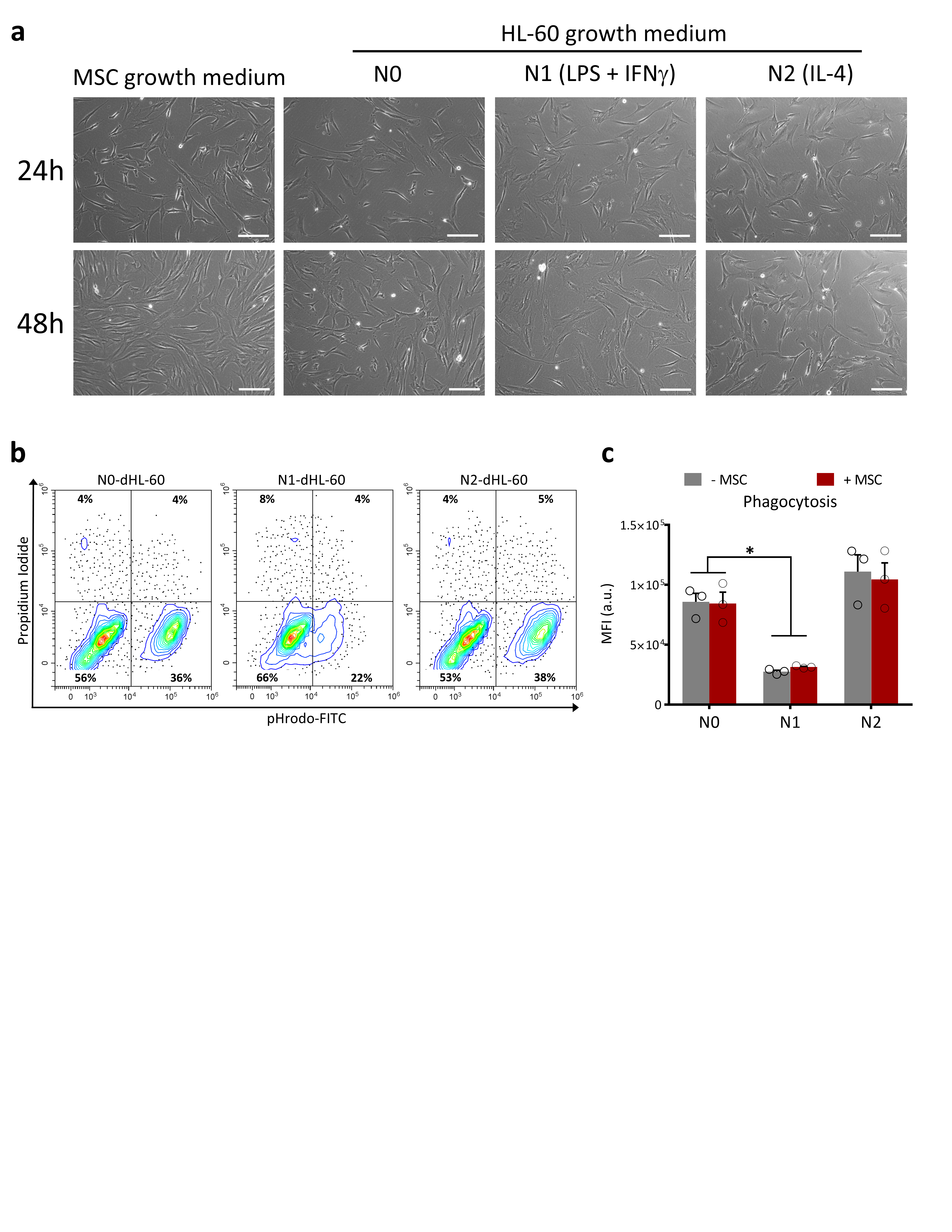

Supplement: Supplementary file 5 — Supplementary Material 5. Fig 4 Transcriptomic analysis of neutrophils. a Bar plot from Venn diagram analysis displaying the overlap of differentially expressed genes (DEGs) in Ly6G⁺ neutrophils isolated at day 3 post-MI from infarcted heart or blood, in mice treated with or without MSCs. The number of unique and shared DEGs between conditions is indicated. b GSEA plot showing the enriched signalling pathways activated in blood neutrophils in response to MSC transplantation. Normalized enrichment scores (NES) are depicted, with statistical significance denoted by the p-value in gradient, and dot size represents gene ratio. c Cell-type enrichment analysis using the Tabula Muris reference signature. Plots show the top significantly enriched immune cell types (Fisher’s exact test, p-adj < 0.05) based on cell origin across the four conditions. [file 13287_2025_4684_MOESM5_ESM.png]
